# Protein Tyrosine Nitration during Development and Abiotic Stress Response in Plants

**DOI:** 10.3389/fpls.2016.01699

**Published:** 2016-11-15

**Authors:** Capilla Mata-Pérez, Juan C. Begara-Morales, Mounira Chaki, Beatriz Sánchez-Calvo, Raquel Valderrama, María N. Padilla, Francisco J. Corpas, Juan B. Barroso

**Affiliations:** ^1^Group of Biochemistry and Cell Signaling in Nitric Oxide, Department of Experimental Biology, Center for Advanced Studies in Olive Grove and Olive Oils, Faculty of Experimental Sciences, University of JaénJaén, Spain; ^2^Group of Antioxidants, Free Radicals and Nitric Oxide in Biotechnology, and Agro-Food, Department of Biochemistry and Molecular and Cellular Biology of Plants, Estación Experimental del Zaidín, Consejo Superior de Investigaciones CientíficasGranada, Spain

**Keywords:** nitric oxide, protein tyrosine nitration, plants, abiotic stress, biotic stress, post-translational modifications

## Abstract

In recent years, the study of nitric oxide (NO) in plant systems has attracted the attention of many researchers. A growing number of investigations have shown the significance of NO as a signal molecule or as a molecule involved in the response against (a)biotic processes. NO can be responsible of the post-translational modifications (NO-PTM) of target proteins by mechanisms such as the nitration of tyrosine residues. The study of protein tyrosine nitration during development and under biotic and adverse environmental conditions has increased in the last decade; nevertheless, there is also an endogenous nitration which seems to have regulatory functions. Moreover, the advance in proteome techniques has enabled the identification of new nitrated proteins, showing the high variability among plant organs, development stage and species. Finally, it may be important to discern between a widespread protein nitration because of greater RNS content, and the specific nitration of key targets which could affect cell-signaling processes. In view of the above point, we present a mini-review that offers an update about the endogenous protein tyrosine nitration, during plant development and under several abiotic stress conditions.

## Introduction

Nitric oxide (NO) is a short-lived gaseous free-radical molecule with high chemical reactivity and diffusion capacity that can mediate most biological actions in which NO is involved. In higher plants, NO plays key roles in several physiological processes and in the response to several biotic and abiotic stress conditions (Beligni and Lamattina, 2000, 2001; Corpas et al., 2008; Chaki et al., 2009a, 2013; Lozano-Juste et al., 2011; Airaki et al., 2012; Begara-Morales et al., 2013; Signorelli et al., 2013; Ziogas et al., 2015; Feigl et al., 2016; Krasuska et al., 2016).

NO is associated with a family of molecules termed reactive nitrogen species (RNS) such as peroxynitrite (ONOO^-^), nitrogen dioxide (NO_2_), dinitrogen trioxide (N_2_O_3_), and such other related molecules as *S*-nitrosoglutathione (GSNO; Corpas and Barroso, 2013a). These RNS have become one of the most noteworthy families of molecules in plant physiology because of their wide range of actions, including signaling processes.

An uncontrolled production of reactive oxygen species (ROS) or RNS can lead to the generation of an oxidative and/or nitrosative stress (Apel and Hirt, 2004; Radi, 2012). In this regard, processes such as lipid peroxidation or protein carbonylation have been widely considered as markers of oxidative stress (Dalle-Donne et al., 2003). However, cell damage can also be mediated by a rise in the RNS levels, thus leading to nitrosative stress (Radi, 2004, 2012). In this respect, mainly under different stress conditions, an overproduction of both ROS and RNS may take place and could mediate damage to biomolecules. A good example of this interaction is the interplay between O_2_^-^ and NO to generate ONOO^-^. This RNS is considered a powerful oxidative agent which, under physiological conditions, can react with CO_2_ and be further decomposed into CO_3_^-^ and NO_2_, a strong nitrating agent (Radi, 2013). In this sense, NO and NO-derived molecules can alter target proteins by post-translational modifications (NO-PTM), with *S*-nitrosylation and protein tyrosine nitration being the most widely studied NO-PTM in plants (Astier and Lindermayr, 2012; Mur et al., 2013; Yu et al., 2014). Protein tyrosine nitration consists of adding a nitro (-NO_2_) group to one of the two equivalent *ortho* carbons of the aromatic ring of tyrosine residues (Gow et al., 2004). This process involves two steps: oxidation of the phenolic ring of tyrosine to tyrosyl radical (Tyr) and the addition of ^⋅^NO_2_ to the Tyr by a nitrating agent. Moreover, the protein tyrosine nitration process could be also modulated by mechanisms of NO crosstalk with ROS scavenging enzymes during abiotic stress tolerance in plants (Arora et al., 2016). Tyrosine nitration is considered a selective process rather than a random one. Furthermore, nitrotyrosine yield is low under physiological conditions, with only 1–5 detectable NO_2_-Tyr residues per 10,000 tyrosines (Bartesaghi et al., 2007). The lower levels of NO_2_-Tyr compared to Tyr content could indicate that protein tyrosine nitration may be a physiological regulator of the signaling pathways in which nitrated proteins are involved. It has also been shown that this PTM is capable of changing the function of a protein by provoking a gain, no effect, or a loss of function, the latter being much more common (Radi, 2004). Currently, protein tyrosine nitration is considered to be an irreversible process. Although some denitrase activities that reverses nitration have been described in mammal cells (Görg et al., 2007; Deeb et al., 2013), a specific denitrase protein has not been identified and no information is available in plants. This PTM seems to be mediated by ONOO^-^, and this indicates that a boost in the number of proteins or an intensification of specific proteins resulting from tyrosine nitration could be considered an indicator of nitrosative stress in plants (Corpas et al., 2007; Corpas et al., 2009a), as has been demonstrated in animal cells. In this respect, **Table 1** summarizes some of the nitrated proteins identified in higher plants together with the nitrated tyrosine residue recognized and the physiological effect of this PTM.

**Table 1:** Examples of proteins identified in higher plants which are targets of tyrosine nitration and the effect of this PTM on their function.

Protein	Effect	Identified nitrated Tyr	Plant Species	Reference
**Stress-related proteins**				
Mitochondrial manganese superoxide dismutase (MSD1)	Decreased activity	Tyr-63^c^	*Arabidopsis*	Holzmeister et al., 2015
Glutathione reductase (GR)	No effect	ND	Pea	Begara-Morales et al., 2015
Monodehydro-ascorbate reductase (MDAR)	Decreased activity	Tyr-345^b^	Pea	Begara-Morales et al., 2015
Dehydro-ascorbate reductase (DAR)	Unknown	ND	Pea	Begara-Morales et al., 2015
Ascorbate peroxidase (APX)	Decreased activity	Tyr-235^b^	Pea	Begara-Morales et al., 2014
**Cytoskeleton proteins**				
α-Tubulin	Mitosis inhibition	ND	Rice and tobacco cell cultures	Jovanović et al., 2010
**Metabolic enzymes**				
Leghemoglobin (Lb)	Inactivation	Tyr-30^b^	Common bean nodules	Sainz et al., 2015
NADP-isocitrate dehydrogenase	Decreased activity	Tyr-392^b^	Pea	Begara-Morales et al., 2013
Hydroxypyruvate reductase (HPR1)	Decreased activity	Tyr-198^c^	Pea peroxisomes	Corpas et al., 2013
Glutamine synthetase	Enzyme inactivation	Tyr-267^c^	*Medicago truncatula*	Melo et al., 2011
Glyceraldehyde-3-phosphate dehydrogenase	Decreased activity	ND	*Arabidopsis*	Lozano-Juste et al., 2011
O-acetylserine(thiol) lyase A1	Decreased activity	Tyr-302^b^	*Arabidopsis*	Álvarez et al., 2011
S-adenosyl homocysteine hydrolase (SAHH)	Decreased activity	Tyr-448^a^	Sunflower	Chaki et al., 2009b
***Proteins involved in Photosynthetic processes***				
Photosystem II protein (PsbO1)	Unknown	Tyr-9^b^	*Arabidopsis*	Takahashi et al., 2015
Carbonic anhydrase (β-CA)	Decreased activity	Tyr-205^a^	Sunflower	Chaki et al., 2013
PSBA(D1) of Photosystem II complex	Disassembly of PSII dimers	Tyr-262^b^	*Arabidopsis*	Galetskiy et al., 2011
Methionine synthase	Decreased activity	Tyr-287^b^	*Arabidopsis*	Lozano-Juste et al., 2011
Ferredoxin–NADP reductase	Decreased activity	ND	Sunflower	Chaki et al., 2011a

*^a^*In silico* identification, ^b^Mass spectrometric techniques (LC-MS/MS), ^c^Site-directed mutagenesis. ND: Not determined.*

**FIGURE 1:**
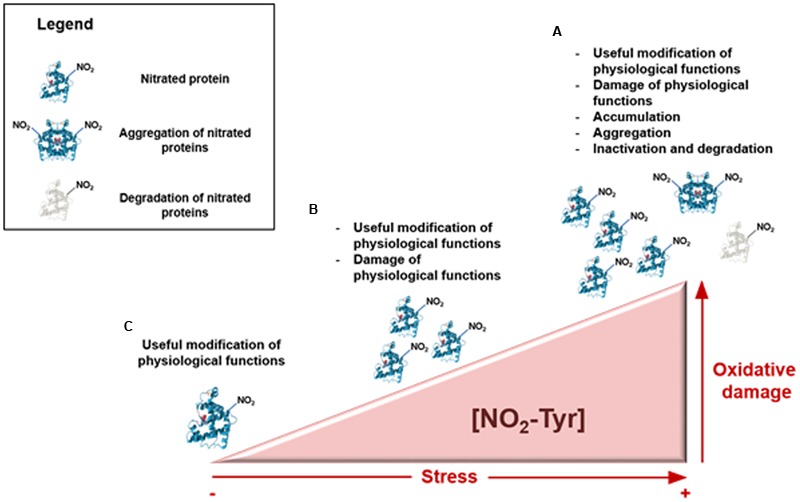
**Time course of protein tyrosine nitration in plant cells under a nitro-oxidative stress.** Depending on the degree of nitro-oxidative stress, three distinct situations can occur. At the beginning of a stress situation **(A)** tyrosine nitration could act as a useful protein modification of physiological functions. When stress become slightly elevated **(B)**, it can provoke the injury of physiological functions and a severe stressful situation **(C)** could trigger accumulation, aggregation and inactivation events.

In addition, plants are exposed to many adverse conditions, which are usually accompanied by a nitro-oxidative stress (Delledonne et al., 1998; Begara-Morales et al., 2014), with a concomitant rise in NO_2_-Tyr content. Stress is even a continuous process and, depending on the degree of stress from regular metabolism and adverse conditions, three key situations can be distinguished (**Figure 1**): regular (**a**), slightly elevated (**b**), and excessive (**c**) degree of stress (Koeck et al., 2005). At the beginning of a stress situation (**a**) and when the stress becomes slightly elevated (**b**), cell machinery including nitration and protein degradation by the proteasome pathway may be designed to cope with these degrees of stress. However, a severely stressful situation (**c**) could provoke the accumulation of nitrated proteins and also protein aggregation with irreversible consequences for the plant. In addition, on certain occasions of increased RNS induced by stress, a rise of protein tyrosine nitration would be expected. Thus, the detection of high protein nitration under a specific stress condition does not necessarily reflect an involvement of tyrosine nitration in stress signaling. In fact, sometimes RNS may increase in proteins prone to nitration. In this respect, it may be important to discern between a widespread protein nitration because of greater RNS content, and the specific nitration of key protein targets which could affect cell-signaling processes. In this sense, we present a mini-review that offers an update about the endogenous protein tyrosine nitration, during plant development and under several abiotic stress conditions.

## Endogenous Protein Tyrosine Nitration and Plant Development

Currently, most investigations on protein tyrosine nitration in plants have focused on the analysis of this process under abiotic stress situations. However, most of these investigations have shown that protein nitration occurs at the physiological level in different plant species. In this regard, it has been shown that ONOO^-^ is endogenously produced in arabidopsis and sunflower (Bechtold et al., 2009; Chaki et al., 2009b) and, very recently, this RNS has been endogenously detected in peroxisomes from arabidopsis plants (Corpas and Barroso, 2013b). These results showing a subcellular location suggest that protein tyrosine nitration could alter the metabolic functioning of these organelles. In this respect, nitroproteome analysis of peroxisomes from pea leaves led to the detection of the endogenous nitration of hydroxypyruvate reductase (HPR1) involved in the photorespiration pathway, the activity of which was inhibited by ONOO^-^ *in vitro* (Corpas et al., 2013). Furthermore, by site-directed mutagenesis it was confirmed that Tyr-198 of Arabidopsis HPR1 is the primary site of nitration responsible for the inhibition of the enzymatic activity by ONOO^-^ and therefore indicating that peroxisomal NO metabolism may contribute to the regulation of physiological processes under no-stress conditions.

On the other hand, it has been noted that protein nitration and NO signaling are clearly involved in plant hormone regulation. In this regard, it has been shown that NO may inhibit abscisic acid (ABA) signaling because NO-deficient plants are hypersensitive to ABA (Lozano-Juste and León, 2010). The *in vitro* nitration of several ABA receptors such as PYR1 and PYL1 by SIN-1 has recently been described (Castillo et al., 2015). This NO-PTM leads to the inactivation of ABA signaling that could be fine tuning ABA-triggered responses. Through mass spectrometry analyses, it was suggested that several Tyr residues could be simultaneously nitrated and hence required for PYR1 nitration-mediated inactivation. PYR1 nitration occurs also *in planta* and it was also shown that nitrated-PYR1 is polyubiquitylated and subsequently degraded by proteasomes. This rapid decrease in ABA responsiveness due to PYR1 nitrated-mediated inactivation could trigger the ABA receptor degradation and thus control ABA signaling (Castillo et al., 2015). Moreover, a rapid NO accumulation has been demonstrated in sunflower hypocotyl protoplasts and adventitious roots (AR) differentiating zone in response to auxin treatment (Yadav et al., 2013).

Other key physiological processes such as photosynthesis have been related to tyrosine nitration processes. The exposure of arabidopsis plants to high concentrations of nitrogen dioxide (NO_2_) and protein analysis by 2D PAGE followed by immunoblot has led to the identification of tyrosine-nitrated protein by mass spectrometry (Takahashi et al., 2015). Among identified proteins, the authors found a selective nitration of the photosystem II (PSII) proteins PsbO and PsbP, and a highly susceptible nitration of four non-PSII proteins including peroxiredoxin II E (PRXII E). Moreover, mass spectrometry analysis identified the Tyr 9 from PsbO as a site for tyrosine nitration. In addition, these results were found under non-physiological NO_2_ concentrations, this set of data suggest that protein tyrosine nitration is a selective mechanism that specifically targets the nitration of a subset of proteins with important roles in plant physiology.

In sunflower hypocotyls, 21 proteins that are immune-reactive against NO_2_-Tyr have been identified these being involved in miscellaneous processes such as photosynthesis and nitrogen metabolism (Chaki et al., 2009b). Moreover, 127 putatively nitrated proteins involved mainly in primary metabolism by LC-MS/MS were identified in Arabidopsis (Lozano-Juste et al., 2011). However, the analysis of nitroproteome in *Citrus aurantium* roots revealed 26 potential proteins to be nitrated (Tanou et al., 2012), showing the high variability in protein nitration among different plant species. Moreover, recently it has been recognized the nitration of different proteins during the ripening of pepper (*Capsicum annuum*), for example the nitration of catalase in red and green and red fruits was very prominent and correlated with the lower catalase activity observed in red fruits (Chaki et al., 2015). Also in pepper plants, it was observed a different protein-nitration profile among radicles, hypocotyls, and cotyledons from this species at different developmental stages (Airaki et al., 2015). This behavior was also prior described in roots of pea plants (Begara-Morales et al., 2013). In that work, an increase in root tyrosine nitration during development was probably due to the general greater NO, ONOO^-^, and ROS content produced during this process. The nitroproteome analysis of 71-day-old pea roots enabled the identification a total of 16 nitrotyrosine-immunopositive proteins by LC-MS/MS, highlighting the nitration of NADP-isocitrate dehydrogenase (ICDH). In this sense, Supplementary Table 1 depicts a summary about the current knowledge on protein tyrosine nitration in some stages of plant development such as germination, juvenility and senescence.

## Protein Nitration Under Adverse Environmental Conditions

Plants are exposed to a plethora of stress conditions and this can seriously compromise crop yield and lead to environmental deterioration. Doubtless, the numerous studies on protein tyrosine nitration in plants have focused on abiotic stress situations and have assumed that a rise in the protein tyrosine nitration is a reliable marker of nitro-oxidative stress (Corpas and Barroso, 2013a). In this regard, all of these studies can provide an overview concerning the relevance of nitration in plant physiology.

### Salinity

One of the major types of abiotic stress affecting plant yield is salinity. The metabolism of RNS in this kind of stress has been studied in different plant species subjected to varying severity of salt stress such as *Olea europaea, Arabidopsis thaliana* and *Citrus aurantium* (Valderrama et al., 2007; Leterrier et al., 2012b; Tanou et al., 2012). In all cases, a general surge in NO_2_-Tyr and ONOO^-^ content was noted together with identification of proteins related to photosynthesis, disease/defense, energy, and storage, among other processes. Otherwise, in roots from 6-day-old arabidopsis seedlings subjected to salinity, an increase of NO and ONOO^-^ production was observed in cytosol, correlating well with the rise in protein tyrosine nitration observed by immunoblot analysis (Corpas et al., 2009b). These findings suggest that salt stress promotes a NO release from peroxisomes to the cytosol for the generation of ONOO^-^, which is involved in protein tyrosine nitration and thus provokes nitrosative stress. Furthermore, in cultures of arabidopsis and tobacco treated with NO donors and exposed to osmotic stress, it has been shown that the cell-wall area is one of the cell components richest in NO_2_-Tyr (Szuba et al., 2015) probably because the highest NO content is located in the cell-wall area. Finally a recent study of 2-day-old sunflower seedlings exposed to 120 mM NaCl has shown an increase in NO_2_-Tyr content in the cells of columella and the peripheral cells in roots (David et al., 2015). This information has been summarized in Supplementary Table 2.

### Extreme Temperatures

Extreme temperature changes are also a major factor limiting plant growth. In this sense, in 3-week-old pea seedlings subjected to high temperature (HT), protein tyrosine nitration increased compared to control values (Corpas et al., 2008). Additionally, the phenomenon of stress generated by a situation of low temperature (LT) displayed a similar pattern, suggesting that these challenges can induce nitrosative stress in pea plants (Corpas et al., 2008). Also, the metabolism of RNS in sunflower hypocotyls exposed to HT was analyzed (Chaki et al., 2011a). A 2.5-fold rise in NO_2_-Tyr content as compared to non-stress plants was detected together with an increase in NO_2_-Tyr and ONOO^-^ content. Notably, a study of nitroproteome identified the induced expression of 13 tyrosine-nitrated proteins related to photosynthesis, carbohydrate, and antioxidant metabolism, with ferredoxin-NADP oxidoreductase (FNR) being distinguished by its *in vitro* inhibition by ONOO^-^ and carbonic anhydrase (CA), whose activity was also inhibited by HT and SIN-1, a peroxynitrite donor (Chaki et al., 2013).

LT stress also affects crop yield and quality. In this respect, leaves from pepper plants exposed to LT for different time periods (1–3 days) showed greater NO_2_-Tyr content after one day of cold treatment, causing nitrosative stress (Airaki et al., 2012). However, after the second and third day of LT, leaves had a lower protein nitration content, indicating that a process of acclimation of pepper plants to LT plants reversed the observed nitrosative stress. Consequently, all these results suggest a direct cross-talk connection between protein tyrosine nitration and stress caused by extreme temperatures.

### Mechanical Wounding

Plants are continuously exposed to agents such as herbivores and environmental mechanical stress that cause wounding and open the way to the invasion by microbial pathogens. With this respect, it has been reported that mechanical wounding increases the NO_2_-Tyr content in all cell types from sunflower hypocotyls (Chaki et al., 2011b). These authors concluded that wounding triggers the accumulation of GSNO and, in a situation of oxidative stress; *S*-nitrosothiols (SNOs) could mediate the process of tyrosine nitration due to ONOO^-^ formation. This could be probably due to GSNO, in the presence of O_2_^-^, is decomposed to radical glutathione (GS^⋅^) and ONOO^-^ and therefore be mediating the observed rise in the content of protein tyrosine nitration. In summary, nitrosative stress is induced in sunflower seedlings and SNOs could act as a new wound signal in plants.

### Heavy Metals

The presence of toxic compounds, such as heavy metals (Cd, Pb, Zn, and Hg) or metalloids (As), can damage plants by altering major plant physiological and metabolic processes. In this sense, exposure of arabidopsis seedlings to arsenic intensified certain immunopositive-nitrated proteins in leaves and prompted a different nitration pattern in the roots of arsenic-treated plants (Leterrier et al., 2012a). Moreover, the sensitivity of different varieties of *Brassica* (*B. napus and B. juncea*) to zinc has also been analyzed. In this sense, both species can accumulate Zn, *B. napus* being the species with higher accumulation of this metal in its organs. This relative Zn tolerance could be related to a distinct alteration of nitration pattern observed by immunoblot (Feigl et al., 2016). Furthermore, under cadmium stress a rise in the ONOO^-^ content in peroxisomes and cytosol from arabidopsis plants has been reported (Corpas and Barroso, 2013b). These results indicate that peroxisomes serve as an endogenous source of ONOO^-^ and that the metabolism of RNS in these organelles could participate in the response to cadmium.

### Water Stress

Currently, studies concerning the analysis of the protein tyrosine nitration process under water stress are very scarce. In this respect, in roots of *Lotus japonicus* plants exposed to water stress, a dramatic rise of tyrosine nitration resulted compared to control (Signorelli et al., 2013). These results suggest that the roots of these plants may be more susceptible to nitration or that the nitration of these proteins could play a role in protection against nitro-oxidative stress. Recently, it has been also reported that NO-pretreatment could prime citrus plants against drought stress (Ziogas et al., 2015). Therefore, these results suggest that this NO-PTM could prepare the plant against a drought stress.

Other type of abiotic stress related to water availability is flooding stress in which the implication of NO has been also studied. Under these anaerobic conditions, nitrite (NO_2_^-^) can accumulate and be used as a substrate by nitrate reductase enzyme for NO generation (Rockel et al., 2002). Therefore, NO could be mediating a protein tyrosine nitration process under these conditions, although this should be further addressed.

## Concluding Remarks

In the last few years, significant advances have been made in understanding the metabolism of NO in plants. However, several challenges regarding the knowledge about the role of nitration as a regulatory element in signaling processes remain to be met or need more thorough research. With respect to protein tyrosine nitration, very few studies have analyzed these phenomena under physiological and different (a)biotic-stress conditions, these situations being insufficiently explored. Moreover, only a few identified nitrated proteins could serve as direct targets for the analysis of signaling mechanisms of NO in plants. Future studies should focus not only on the analysis of protein tyrosine nitration as a nitro-oxidative marker but also on how this NO-PTM affects to the modulation of key proteins in particular cell-signaling processes in plants. Therefore, further research will be necessary to discover the pathways linking ROS, RNS, and (a)biotic stresses.

## Author Contributions

JB and CM-P wrote the article; CM-P, JB-M, MC, BS-C, RV, MP, FC, and JB have revised the manuscript.

## Conflict of Interest Statement

The authors declare that the research was conducted in the absence of any commercial or financial relationships that could be construed as a potential conflict of interest.
