# Supplementary material for: Protein Tyrosine Nitration during Development and Abiotic Stress Response in Plants
Source: Front Plant Sci. 2016 Nov 15;7:1699. doi: 10.3389/fpls.2016.01699 (PMC5108813; doi:10.3389/fpls.2016.01699)
Supplement: Supplementary file 1 [file Table_1.PDF]

| **Plant Stage** | Germination 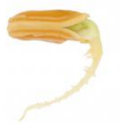 | Juvenility 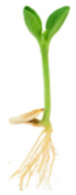 | | | Senescence 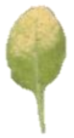 |
|---|---|---|---|---|---|
| **Plant Age** | After 24h of imbibition | 9-day-old hypocotyls | 14-day-old seedlings | 7-, 10- and 14-day-old radicle, hypocotyl and cotiledons | 71-day-old plants |
| **Plant Species** | *Malus domestica* Borkh (Apple) | *Helianthus annus* (Sunflower) | *Arabidopsis thaliana* | *Capsicum annuum* (Pepper) | *Pisum sativum* (Pea) |
| **RNS Metabolism and technical approach** | ↓ $NO_2$-Tyr (Immunoblot) in soluble protein fraction of root axis | 21 immunoreactive proteins against $NO_2$-Tyr (LC-MS/MS) | Identification of 127 putatively nitrated proteins (LC-MS/MS) | Decreasing intensity of immunoreactive proteins in roots and hypocotyls with plant age | ↑ $NO_2$-Tyr (Immunoblot) in pea roots and identification of 16 nitrotyrosine-immunopositive proteins (LC-MS/MS) |
| **References** | *Krasuska et al., (2016)* | *Chaki et al., (2009b)* | *Lozano-Juste et al., (2011)* | *Airaki et al., (2015)* | *Begara-Morales et al., (2013)* |

**Supplemental Table 1. Plant developmental processes affected by tyrosine nitration.** Selected stages of plant development have been depicted in this table illustrating the main results derived from each study regarding protein tyrosine nitration process.
