# Supplementary material for: Protein Tyrosine Nitration during Development and Abiotic Stress Response in Plants
Source: Front Plant Sci. 2016 Nov 15;7:1699. doi: 10.3389/fpls.2016.01699 (PMC5108813; doi:10.3389/fpls.2016.01699)
Supplement: Supplementary file 2 [file Table_2.PDF]

Plant species	Plant age	Type of salt stress	RNS metabolism	References
Olea europaea	45-day-old plants	200 mM NaCl	↑ NO_2-Tyr (Immunoblot)	*Valderrama et al., (2007)*
Arabidopsis thaliana	6-day-old seedlings	100 mM NaCl	↑ $ONOO^-$ (CLSM)	*Leterrier et al., (2012b)*
Citrus aurantium	172-day-old plants	150 mM NaCl	↑ NO_2-Tyr (Immunoblot)	*Tanou et al., (2012)*
Arabidopsis thaliana	6-day-old seedlings	100 mM NaCl	↑ $ONOO^-$ (CLSM)<br>NO_2-Tyr (Immunoblot)	*Corpas et al., (2009b)*
Arabidopsis thaliana and Nicotiana tabacum	Cell suspensión cultures	190 mM NaCl	↑ NO_2-Tyr (CLSM and immunoblot)	*Szuba et al., (2015)*
Helianthus annuus	2-day-old seedlings	120 mM NaCl	↑ NO_2-Tyr (CLSM and immunoblot)	*David et al., (2015)*

Supplemental Table 2. Summary table of protein tyrosine nitration metabolism under salt stress in different plant species. CLSM: Confocal Laser Scanning Microscopy**.**
